# *μ*-FTIR, *μ*-Raman, and SERS Analysis of Amide I Spectral Region in Oral Biofluid Samples during Orthodontic Treatment †

**DOI:** 10.3390/s22207874

**Published:** 2022-10-17

**Authors:** Carlo Camerlingo, Marianna Portaccio, Fabrizia d’Apuzzo, Ludovica Nucci, Letizia Perillo, Maria Lepore

**Affiliations:** 1 CNR-SPIN, Consiglio Nazionale delle Ricerche, Istituto Superconduttori, Materiali Innovativi e Dispositivi, 80078 Pozzuoli, Italy; 2 Dipartimento di Medicina Sperimentale, Università della Campania “L. Vanvitelli”, Via S. Maria di Costantinopoli 16, 80138 Napoli, Italy; 3 Dipartimento Multidisciplinare di Specialità Medico-Chirurgiche e Odontoiatriche, Università degli Studi della Campania Luigi Vanvitelli, 80138 Napoli, Italy

**Keywords:** *μ*-FTIR, *μ*-RS, SERS, Amide I, gingival crevicular fluids, orthodontic processes

## Abstract

Gingival crevicular fluid (GCF) is a site-specific exudate deriving from the epithelium lining of the gingival sulcus. GCF analysis provides a simple and noninvasive diagnostic procedure to follow-up periodontal and bone remodeling in response to diseases or mechanical stimuli such as orthodontic tooth movement (OTM). In recent years, the use of vibrational spectroscopies such as Fourier Transform Infrared and Raman microspectroscopy and Surface-Enhanced Raman spectroscopy contributed to characterizing changes in GCF during fixed orthodontic treatment. Amide I band plays a relevant role in the analysis of these changes. The aim of this study was to investigate the spectroscopy response of Amide I depending on the OTM process duration. A model based on Gaussian–Lorentzian curves was used to analyze the infrared spectra, while only Lorentzian functions were used for Raman and SERS spectra. Changes induced by the OTM process in subcomponents of the Amide I band were determined and ascribed to secondary structure modification occurring in proteins. The vibrational spectroscopies allow us to efficiently monitor the effects of the orthodontic force application, thus gaining increasing attention as tools for individual patient personalization in clinical practice.

## 1. Introduction

**Figure 1:**
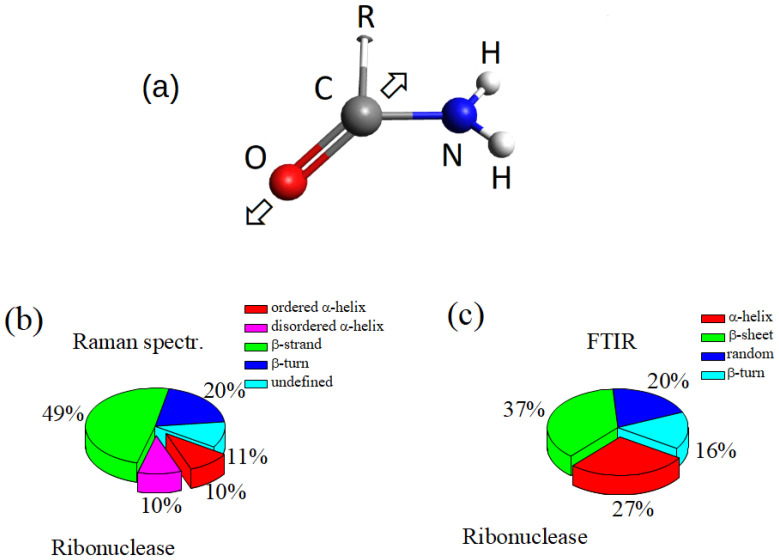
(**a**) Sketch of the Amide I functional group. “R” represents a not specified of organic group; the arrows indicate the bond vibration direction; (**b**) relative distribution of the secondary structures of the Raman response of Ribonuclease in the Amide I region [14]; (**c**) relative distribution of the secondary structures of the FTIR response of Ribonuclease in the Amide I region [16].

The joint use of complementary analytical techniques is a fundamental aspect of the current approach to investigate biological fluids and matter. Among a large number of physical and chemical assessment techniques, spectroscopy-based methods are gaining increasing importance thanks to technological progress and the growing knowledge of inherent phenomenology. In this work, we used Fourier Transform Infrared and Raman microspectroscopy (μ-FTIR and μ-RS), and Surface-Enhanced Raman Spectroscopy (SERS) for investigating human gingival crevicular fluid (GCF) samples, as preliminarly reported in a communication at the 8th Electronic Conference on Sensors and Application (ECSA 8) [1]. Both μ-FTIR and μ-RS techniques allow us to extract information on chemical bonds and secondary protein structure from very small quantities of the sample [1,2,3,4,5]. However, the involved mechanisms of these two techniques are different, and complementary information can be obtained. In fact, FTIR is based on light absorption and is related to the transition dipole moment of molecular electrons, while Raman depends on electrical polarizability. FTIR has a high sensibility and is extensively used for molecular characterization [6]. Actually, two drawbacks of FTIR are the influences on the signal of O–H contribution originating from water or water vapor, and the often occurring overlapping of peaks. Raman spectroscopy is less sensitive to water and, in general, allows a better spectral resolution, even if the sensitivity is lower. Furthermore, micro-Raman spectroscopy has a high spatial resolution, allowing us to sense any inhomogeneities in the sample composition. Moreover, SERS can allow a significant enhancement of Raman signal that is generally low for biological fluids [7]. The GCF is a fluid deriving from the epithelium lining of the gingival sulcus. The GCF contributes to the host defense of the periodontal space by flushing bacterial colonies and their metabolites away. It has a complex composition that includes blood electrolytes and many organic molecules such as albumins, globulins, lipoproteins, leukocytes, neutrophils, and lymphocytes. A comprehensive proteomic analysis of GCF has been reported by L.H. Ngo et al. [8], who identified many different proteins and peptides. The GCF composition reflects the current state of the periodontal site, allowing the monitoring and diagnosting of periodontal pathological states and gingivitis [9,10]. The tooth repositioning by orthodontic force application modifies the GCF composition due to the release of chemical substance cascades generated by the activation of many complex cellular and molecular mechanisms [3,5]. The abovementioned spectroscopic methods allowed us to assess the changes occurring in the main functional molecular groups through the analysis of spectroscopy response in the skeletal, Amide I, and Amide III regions [3,5]. The changes are significant and can be used to monitor and classify patient state, as conducted in Ref. [3] where the Principal Component Analysis (PCA) provided a reliable statistical method for analyzing spectroscopy data. As we observed in previous works [2,3,4,5], particularly relevant GCF changes seem to be related to the modifications of the secondary configurations of hosted proteins resulting from the complex response of the proteins to the stress of the orthodontic process. For this reason, the analysis of the Amide I region in vibrational spectra can be particularly interesting because it plays a pivotal role in the characterization of protein secondary structure ([11,12,13,14,15] and references therein). The spectrum of the Amide I region is related to C=O bond vibrations (Figure 1a). The associated energies critically depend on the secondary structure of the protein where the bond is placed, originating a band of vibrational modes centered on several energies that are assigned to different secondary structures of the protein. In the Ref. [14], R.W. Williams individuated the main secondary structures from the Raman response of protein, namely α-helix, $3_{10}$-helix, β-strands to form β-sheets, β-bridge, β-turn, and disordered coils, respectively. The relative intensity of these modes depends on the specific proteins, but for a large number of cases, the 20% contribution is due to α-helix (in the ordered and disordered state), about 50% are related to unfolded β-structure, and the remaining part, to turn, to disordered configurations, as schematically reported in Figure 1b, of the Ribonuclease protein [14]. In Figure 1c, the relative distribution of the modes in the Amide I band of ribonuclease protein from the FTIR data is shown [16]. In this case, the peak area of α-helix is about 27% of the total area of the Amide I band, β-sheet and β-turn contribute 37% and 16%, respectively, and the remaining 20% was assigned to random configurations [16]. The secondary structure of proteins has been generally evaluated by analyzing the Amide I region of the FTIR spectrum, but in recent years, μ-RS has been also fruitfully considered for this aim due to the reduced interference with the water signal [13]. Then, it is expected that a joint use of μ-FTIR, μ-RS, and SERS could improve the analysis of the Amide I region. In the present work, we analyzed and compared the Amide I region spectra obtained by μ-FTIR and μ-RS spectroscopy of GCF samples that were collected from informed subjects during orthodontic processes for tooth adjustment by metallic tensors springs. The GCF was probed before the orthodontic treatment started and after 2, 7, and 14 days of the clinical process. SERS measurements were also performed on these GCF samples in order to take advantage of the signal enhancement effects.

## 2. Materials and Methods

### 2.1. Subjects

The GCF samples were collected before and during different time points of orthodontic treatment with fixed appliances treated at the Orthodontic Program of the University of Campania “Luigi Vanvitelli” (Naples, Italy). Informed patients aged between 12 and 22 years were included in this research after the approval of the Ethics Committee of the University. The patients were all Caucasian (13 males and 5 females) according to these selection criteria: (1) dental malocclusion needing fixed orthodontic treatment in permanent dentition; (2) no previous orthodontic treatment; (3) good general and periodontal health; and (4) no drug assumption at least 1 month before the beginning of the study. Conventional metal orthodontic brackets (MBT${}^{\mathrm{TM}}$; 3M United; Monrovia; Cali, CO, USA) were applied on the buccal surface of upper and/or lower permanent teeth, and 0.014 inches NiTi archwire with elastic ligatures were used. Further details on patient selection criteria and clinical methods are reported in previous Ref. [4].

### 2.2. Collection of Gingival Crevicular Fluid

The GCF samples were collected by standardized sterile absorbent paper cones. They were inserted 1 mm into the gingival crevice and left in situ for 30 s. Two cones were consecutively used (60 s of interval) to maximize GCF volume per site. This operation was performed at different times along the orthodontic process, namely before bracket bonding (T0) and after 2 (T1), 7 (T2), and 14 (T3) days of treatment. Paper cones were transferred to sterile plastic vials and stored at $-80^{\circ }$. For Raman measurements, GCF samples were examined directly in the paper cones. For μ-FTIR and SERS measurements, the fluid was extracted from cones adding 10 mL of distilled water and was vortexed for 30 s and centrifuged (10 min, 800× *g*).

### 2.3. μ-FTIR Spectroscopy

A Multiscope system infrared microscope equipped with a Perkin Elmer Spectrum One FT-IR spectrometer was used to acquire FTIR spectra. A few microliters of fluid sample was dropped on an aluminium IR-reflective surface and left to dry. The acquisition was performed in transflection mode, subtracting the background from signal due to the metallic IR-reflective surface. Spectra were acquired from different sampling spots (100 × 100 μm^2^) on the surface of the drops. All spectra were collected using 64 scans in the range 4000 to 600 cm${}^{-1}$ with a 4 cm${}^{-1}$ spectral resolution. Further details are reported in Ref. [4].

### 2.4. μ-RS and SERS

The μ-RS and SERS of GCF samples were performed by using a micro-Raman system working in the visible regime, equipped with a Jobin-Yvon TriAx 180 monochromator, a liquid N${}_{2}$-cooled CCD, and a grating of 1800 grooves/mm allowing a spectral resolution of 4 cm${}^{-1}$. The excitation source was a He–Ne laser operating at a wavelength λ = 633 nm with a maximum nominal power of 17 mW. The laser beam was focused on the surface of the GCF soaked paper cone by a 100× optical objective (n.a. = 0.90). Spectra were acquired from different regions of the cones using a spot area of about 5 μm of size. The spectra were obtained using accumulation times ranging from 60 to 300 s; μ-RS was also performed on a pristine paper cone (without GCF) under experimental conditions similar to those used for sampling GCF to distinguish the signal from the substrate contribution and subtract it from data. For SERS measurements, gold nanoparticles (GNP) were prepared by a citrate reduction method consisting of a reduction process of a 0.01% HAuCl${}_{4}$ water solution by 1% sodium citrate [17,18]. The final GNP diameter was determined by the added amount of sodium citrate. A detailed description and image and structural characterization of the GNP fabrication process is reported in Ref. [18]. The characterization included absorption spectroscopy which obtains information on the plasmon resonance peak [19,20]. Optimal conditions for SERS were achieved by GNP with a diameter of about 30 nm. SERS spectra were collected using a 50× (n.a. = 0.75) objective and an accumulation time of 300 s. A microliter drop of the obtained GNP colloid was placed on a microscope glass and left to dry for some hours. A small amount (5 μL) of the GCF sample was then deposited on the microscope glass decorated with dried GNP preparation and immediately after SERS measurements were performed. In this case, different regions of the drops were also used for acquiring spectra.

### 2.5. Data Analysis

The data obtained from Raman and FTIR spectroscopy were analyzed in a similar way. The raw spectra were preliminarily normalized to the deviation from the media value using the Standard Normal Variate (SNV) method. The normalized signal intensities $y_{N}$ were calculated from the spectral data *y* using the following relation:

(1) $$y_{N}=\frac{y}{{\left[\frac{1}{N-1}\sum {\left(y-<y>\right)}^{2}\right]}^{1/2}}$$

where <*y*> is the average value and *N* the number of data points. In this way, the standard deviation of $y_{N}$ results equal to 1. Background signal was subtracted from the spectrum by using a wavelet algorithm [21] implemented in Python framework using the open source PyWavelets packet [22]. A Direct Wavelet Transform (DWT) was performed at the 6th level using biorthogonal “bior6.8” wavelet function. Using the calculated coefficients, the signal was reconstructed, neglecting the 6th level approximation coefficients. For the specific case of the analysis of GCF on the cellulose substrate, the signal from the cellulose substrate was subtracted using a linear regression of the data with the signal of the bare substrate.

For showing the similarities between spectra acquired from samples in the same experimental conditions and highlighting the differences between spectra from GCF samples collected in different stages of orthodontic treatment, a univariate analysis that was already adopted in Refs. [4,23] was used. For each wavenumber point, the intensity of the spectrum ($y_{i}$ variable) was compared to the corresponding value of another spectrum variable ($x_{i}$ variable) referring to a different sample by performing a linear regression or univariate analysis of data. The spectrum Y (containing the n data $y_{i}$) was assumed to be a linear function of another spectrum X (containing the n data $x_{i}$), that is $y_{i}$ = ($my_{i}+p+\varepsilon_{i}$). If there are no structural changes in the sample, then the perturbation term $\varepsilon_{i}$ is due only to the experimental conditions and follows a Gaussian distribution, with the mean equal to zero. Regression analysis enables the evaluation of the parameters *m* and *p*. To estimate the similarity between data sets, the sample coefficient of determination $R^{2}$ can be evaluated. It is defined as:

(2) $$R^{2}=1-\frac{\sum {\left[y_{i}-\left(mx_{I}+p\right)\right]}^{2}}{\sum {\left[y_{i}-<y>\right]}^{2}}$$

where <*y*> is the average value of vector Y. $R^{2}$ ranges from 0 for uncorrelated data to 1 for perfect linear dependence. The linear regression was evaluated for a number of points ranging between 500 and 700, suggesting a high level of significance of the procedure. This analysis was performed with MATLAB (Version 8.3, Math-Works, Natick, MA, USA) program.

The main modes that contribute to the Amide I signal were determined by fitting the spectrum with a model obtained by the sum of elemental Lorentzian and/or Gaussian functions in the 1580–1700 cm${}^{-1}$ spectral region. We used a best-fit peak-fitting routine of GRAMS/AI program (2001, Thermo Fisher Scientific, Waltham, MA, USA), based on the Levenberg–Marquardt nonlinear least-square method. The main peaks were manually selected in order to define the starting conditions for the best-fit procedure. The bestfit was then performed to determine the envelope of peaks with optimized intensities, positions, and widths, adopting $\chi^{2}$ parameter as the performance index. The choice of Lorentzian or Gaussian shape for the mode components of the model depends on the spectroscopy methods [24]. Basically, Lorentzian function is considered to appropriately shape the spectroscopy modes. This assumption is based on a straightforward analogy between the bond vibration and the dissipative motion of a harmonic oscillator driven by a sinusoidally oscillating force. In the case of Raman spectroscopy, the Lorentzian function adequately represents the observed signal, but, for FTIR spectra, a Gaussian shape is often more suitable to model the experimental data. As discussed in Ref. [24], these features are generally related to a broadening of the intrinsic signal. The physical interaction with adjunctive effects or the influence of the measurement system induces a broadening of the spectroscopic signal that can be accounted for by a Gaussian shape. This more frequently occurs in FTIR spectra, and then Gaussians are preferred for modeling the vibrational modes in these cases [15,25]. Components, either Lorentzian- and Gaussian-shaped, were considered for determining a good model of the experimental FTIR spectrum. One-way ANOVA test with a 0.05% or 0.01% significance level was used for the statistical analysis of the differences among the considered data sets.

## 3. Results

### 3.1. Univariate Analysis Results

The $R^{2}$ parameter was evaluated for estimating the correlation between vibrational spectra acquired from GCF samples using the same spectroscopic techniques and collected at the same stage of the orthodontic treatment. Its value ranged between 0.99 and 0.96, thus indicating a high overall correlation. Conversely, the $R^{2}$ values estimated for spectra from samples collected at different stages of the orthodontic treatment were significantly lower, ranging from 0.75 to 0.82. After checking the results of the univariate analysis, we used the average spectra (evaluated by considering all the spectra obtained for samples in a given experimental condition) for all the further analysis reported in the present paper.

### 3.2. μ-Raman Spectroscopy

**Figure 2:**
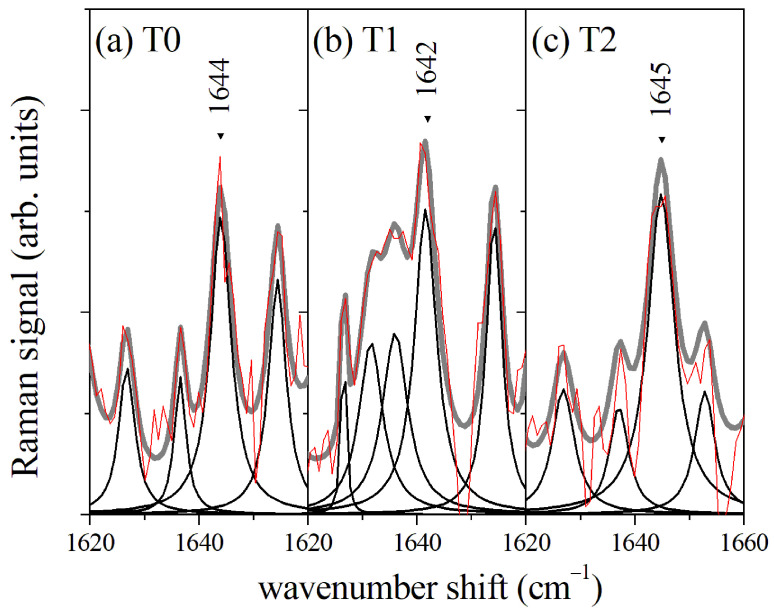
(**a**) Average Raman signal of GCF from control samples before starting the orthodontic process (T0); (**b**) Average Raman spectrum of GCF from samples collected after 2 days of orthodontic process (T1); (**c**) Average Raman spectrum of GCF from samples collected after 7 days of orthodontic process. The red lines refer to the measured signal and are compared with the envelope (gray line) of evaluated mode components (black lines).

In a first approach, μ-RS of GCF samples were performed directly on the cellulose cones used for collecting the fluid from the gingival sulcus, without implementing any extraction process. The signal due to the cellulose substrate was subtracted by a suitable numerical data treatment based on the wavelet algorithm [21]. The average Raman spectrum in the Amide I region collected before starting the orthodontic process (T0) is reported in Figure 2a. Lorentzian functions were used to model the Raman modes. The spectrum was dominated by the Amide I component assigned to α-helix secondary structure mode at a wavenumber value of 1644 cm${}^{-1}$. The position of the α-helix mode did not change significantly during the orthodontic process, as evidenced in Figure 2b,c for GCF collected after 2 days (T1) and 7 days (T2) of orthodontic tooth movement (OTM), respectively. The main Raman modes in the Amide I spectral region are indicated in Figure 2 and listed in Table 1. The mean value of the areas of the spectra and their standard deviation are reported for each Raman mode. The area values were normalized to the whole area of the Amide I band and reported as a percentage. The significance level was estimated by the one-way ANOVA test between the data referring to T1 and T0 samples and between those referring to T2 and T1 samples. In agreement with the literature on protein spectroscopy [3,5,14], we assigned the mode at 1644 cm${}^{-1}$ to α-helix secondary structure, the modes at 1618 cm${}^{-1}$ and 1675 cm${}^{-1}$ to β-sheet, the mode at 1665 cm${}^{-1}$ to β-turn, and the mode at 1654 cm${}^{-1}$ to random coil, respectively. An overlap of $3_{10}$-helix and β-sheet modes at about 1620 cm${}^{-1}$ is expected, thus this assignment cannot be unequivocal. The Raman spectrum referring to GCF collected after 2 OTM days (T1, Figure 2b) showed the largest difference with the spectra acquired before (T0) and after 7 days (T2) of OTM, respectively. The intensity of β-sheet mode at 1675 cm${}^{-1}$ increased with respect to the α-helix contribution, proving the likely occurrence of protein unfolding processes. When the OTM duration increased to 7 days, a partial recovering of the secondary structure configuration of the proteins occurred, and the spectrum for T1 data was qualitatively closer to the Raman response of the GCF before OTM. An μ-RS was also performed on GCF extracted from the paper cones. The collected spectra did not differ significantly from the data obtained from the direct acquisition on the paper cones.

### 3.3. μ-FTIR Spectroscopy

**Figure 3:**
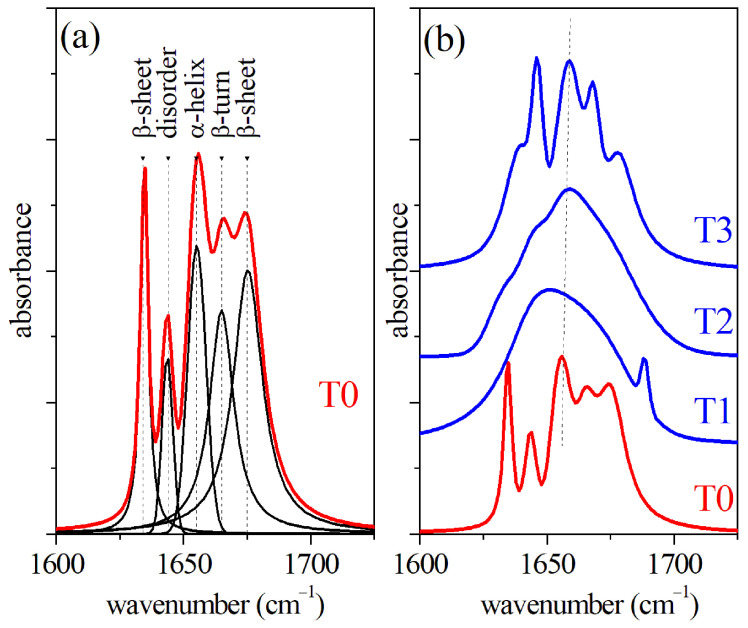
(**a**) FTIR spectrum of GCF collected before OTM (T0) and results of the deconvolution procedure; (**b**) comparison of FTIR spectra of GCF collected before OTM (T0) and after 2 days (T1), 7 days (T2), and 14 days (T3) of the OTM process.

The μ-FTIR spectroscopy also provides a suitable and sensitive method to analyze GCF [4,5,26,27]. The Amide I region of the FTIR spectra obtained from dried samples of GCF are reported in Figure 3. The spectra refer to GCF samples collected at different times of the OTM process, namely before the treatment (T0) and after 2 days (T1), 7 days (T2), and 14 days (T3) of OTM, respectively. In Figure 3a, the FTIR spectrum of GCF before OTM is reported. The main modes occurring in the spectrum have been determined by modeling the signal in terms of Lorentzian and Gaussian functions. The different mode positions are reported in Figure 3a. In particular, β-sheet (1634 cm${}^{-1}$), disordered components (1644 cm${}^{-1}$), and α-helix (1655 cm${}^{-1}$) secondary structure of the protein contributions were determined [28]. The precise values of mode positions and relative peak area for the four OTM steps are reported in Table 2.

The values of the peak area were normalized to the whole Amide I band area and are reported as a percentage. In this case, the mean value of the areas of the spectra and their standard deviation are also reported for each absorption peak. The significance level was estimated by the one-way ANOVA test between the data referring to T1 and T0 samples, T2 and T1, and T3 and T2, respectively. The T0 spectrum is also reported in Figure 3b and compared with spectra obtained from GCF samples collected after the T1, T2, and T3 OTM steps, respectively. A significant smearing of the α-helix mode peak of μ-FTIR the spectrum was clearly visible during the first week of the OTM process (curves T1 and T2 of Figure 3b). A partial recovery was noticed in the last part of the OTM (T3) where the α-helix mode returned sharp. It should also be noted that in the T2 spectrum, the mode assigned to disordered component increased in intensity and, in general, the area of peak assigned to β-sheet component was larger than the value estimated for T0 case all along the OTM period. A change of relative weight of the components was also observed for Raman spectra during the OTM, reflecting a change of the secondary structures of the proteins. As in the case of FTIR, the β-sheet component area increased with OTM time, probably due to the occurrence of unfolding processes in the proteins. Apparently, the α-helix mode of the Raman spectra looked weakly affected by the OTM, and both the intensity and position did not change significantly. The helical structure of the protein provides an enhanced resistance to mechanical strain, prevents breakage, and withstands large deformations [29,30]. In terms of Raman response, we expected a partial transition from α-helix to β-sheet conformations, promoted by hydrogen relocation, and an increase of disorder [29,30].

### 3.4. SERS Measurements

**Figure 4:**
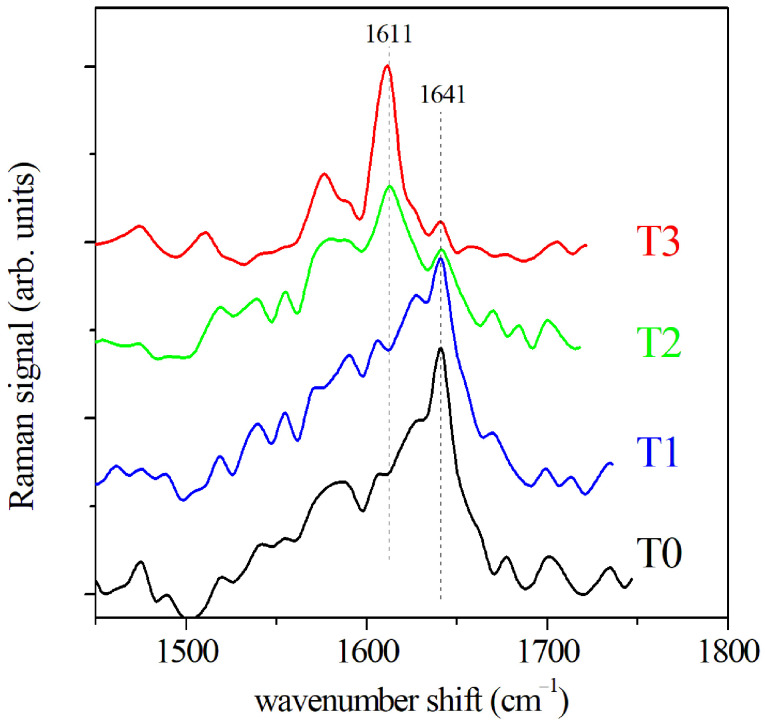
SERS spectra of GCF before OTM (T0) and at increasing OTM times, namely 2 days (T1), 7 days (T2), and 14 days (T3), respectively.

The GCF samples extracted from paper cones were also tested by SERS. The SERS spectra of the Amide I region are reported in Figure 4 for the GCF samples collected before the OTM and after 2, 7, and 14 days of OTM, respectively. Only the regions of the fluid in the proximity of the gold nanoparticle surface contribute to SERS signal, thus this one depends critically on the conformation of the molecule samples in these regions, and it allows us to obtain spatially resolved information on the secondary structure of the proteins. A shift of Amide I band is clearly visible in the SERS spectra shown in Figure 4. The maximum of the signal intensity moves from 1641 cm${}^{-1}$ to 1611 cm${}^{-1}$, increasing with OTM time. This is due to a change of the mode distribution, in particular concerning the α-helix mode. The α-helix mode is characterized by a structure having 3.6 residues/turn and it is, typically, the predominant helix configuration in proteins. However, a helical configuration shorter than the usual α-helix with three residues/turn ($3_{10}$-helix) can also occur [31]. It is presumed to act as a intermediate step for α-helix formation and folding favored by a reciprocal low barrier energy [32]. The energy of $3_{10}$-helix SERS mode was lower than the α-helix mode [33,34], and we assigned to it the observed 1611 cm${}^{-1}$ SERS peak. Only the helix components of the Amide I band significantly contribute to the SERS signal. Due to the local character of the SERS mechanism, the signal is mainly generated by vibration of the molecular bonds close to the gold nanoparticle surface. For this reason, a regular structure, as helix-ones, are favored over other types of components as indicated by Kurouski et al. [35].

### 3.5. Comparison of μ-FTIR and μ-RS Amide I Spectra

**Figure 5:**
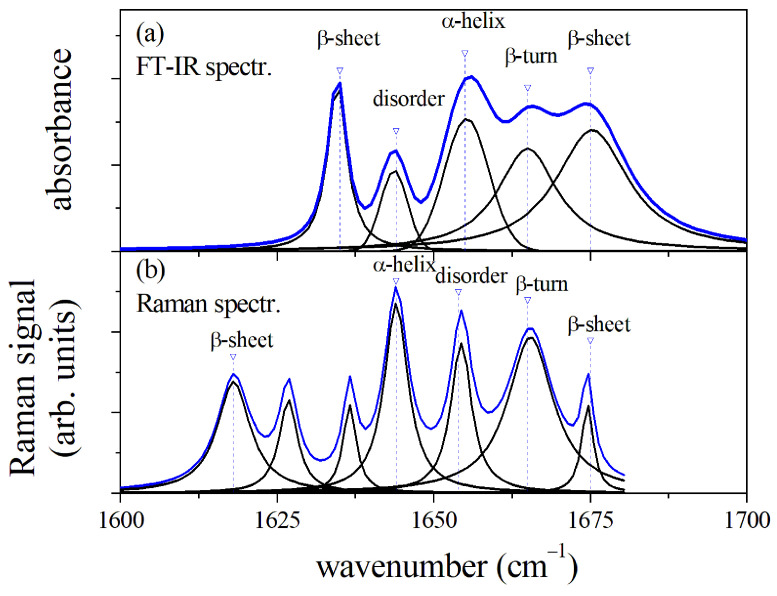
Comparison of μ-FTIR (**a**) and μ-RS Amide I spectral region (**b**) of GCF before OTM (T0). The position of mode components and assignments are reported for the two spectra.

Both μ-FTIR and μ-RS spectroscopies are valuable methods to investigate the secondary configuration of proteins in organic fluids through the analysis of the spectral region of Amide I, as we have shown for the GCF case. However, differences and peculiarities of the two methods have to be taken into account to make a correct interpretation of the spectroscopy data. A direct comparison of the μ-FTIR and μ-RS spectra in the Amide I region allows us to make this point. In Figure 5, the spectra of GCF from μ-FTIR (Figure 5a) and μ-RS (Figure 5b) spectroscopies are reported and compared in the spectral range of 1600–1700 cm${}^{-1}$. The main modes of the two spectra were determined by fitting the curves by a sum of Lorentzian or Gaussian functions as described above, and their assignments were reported. Both the spectra are dominated by the the α-helix mode, but the spectral position of this mode is different, occurring at about 1652 cm${}^{-1}$ for the μ-FTIR spectrum and at 1644 cm${}^{-1}$ for the Raman signal. Consequently, the spectral weight of the Amide I band was influenced, resulting in a higher wavenumber in the case of FTIR [36]. It is also relevant to note that the positions of the mode attributed to disordered states are different for the two spectra, resulting at lower or higher wavenumber with respect to the α-helix mode center for μ-FTIR and μ-RS, respectively [14,16]. This aspect can help to correctly shape the molecular state of proteins and determine any changes by comparing the two methods. Similarly, the higher spectral resolution of μ-RS can provide a guide to correctly deconvolve the μ-FTIR signal. Specifically, we saw the possibility given by SERS to overcome the mode overlapping between $3_{10}$-helix and β-sheet at about 1620 cm${}^{-1}$ and determine the formation of $3_{10}$-helices in GCF after OTM.

As previously stated, μ-FTIR and μ-RS Amide I spectral analysis indicates the probable occurrence of unfolding processes during the initial stage of OTM and a certain restoration in the following days. This behaviour can be related to the properties of the helical protein structure and its response to strain forces, largely considered in the literature, both theoretically [29] and experimentally [30,37]. Thanks to the unique helical structure, enhanced resistance to mechanical strain is a protein characteristic that prevents the occurrence of breakage and large bonding deformations. The basic mechanism of protein unfolding of the α-helix is due to a reversible breaking of H-bonds. Mechanical energy can be dissipated thanks to these processes through the formation of specific regions, and the breakage of stronger molecular bonds can be avoided [30]. When a strain is applied, it induces stress for beginning the modification of the α-helix structure or the transformation from the α-helix to the β-sheet conformation. This can be considered as an unfolding–refolding process that causes hydrogen relocation and an increase of disorder [2,37]. It can be interesting to note that the changes occurring in the Amide I spectral region could also be ascribed to the formation of amyloid aggregates that can be induced by the mechanical stress that is present during OTM. Some researchers describe the potential role of mechanical stress in a pathway underlying the mechanisms of chronic inflammation, cancer, and Alzheimer’s disease [38,39]. All these pathologies are characterized by the presence of amyloid aggregates. Since inflammation processes are also present during OTM, a potential role of amyloid can be envisaged also in the present case. This perspective, therefore, requires further analysis of the spectral region of the Amide I, since this can provide useful information in this regard [40,41].

## 4. Conclusions

The joint use of different vibrational spectroscopic techniques enables us to study the Amide I region in a more detailed way; the μ-RS technique enables the study of GCF samples during OTM without any sample manipulation. The spectra can be directly acquired from the paper cones used for collecting the biofluid. The extraction of GCF provides samples that can be investigated using μ-FTIR and SERS methodology. Both μ-FTIR and μ-RS Amide I spectral region analysis can indicate the occurrence of unfolding processes during the first 7 days of OTM and a partial recovery during the following 7 days. Both μ-RS and SERS allow us to characterize samples with a better spatial resolution in comparison with μ-FTIR, allowing us to distinguish between different α-helix conformations, namely α-helix and $3_{10}$-helix structures in a more precise way. In fact, the correspondent modes in μ-FTIR and μ-RS spectroscopies are not distinguishable due to the energy overlap. In the present case, SERS indicate an increase of $3_{10}$-helix components that can demonstrate the capacity of the system to regenerate after the orthodontic stress and reconstruct the protein environment. As said before, alternative mechanisms related to the formation of amyloid-type aggregates should also be considered, and a further analysis of Amide I spectral region could allow a better description of the processes occurring during OTM.

## Figures and Tables

**Table 1:** Wavenumber position and relative peak areas of the main modes of μ-RS Amide I spectral region of GCF and their assignment. The GCF were collected before starting the orthodontic process (T0 data) and after 2 days of treatment (T1 data) and 7 days of treatment (T2 data). The peak area percentage was evaluated by a fitting procedure, assuming the Amide I band area equal to 1. The area values are shown as ‘mean ± standard deviation’ for each group.

T0 Modes (cm${}^{-1}$)	T0 Peak Area (%)	T1 Modes (cm${}^{-1}$)	T1 Peak Area (%)	T2 Modes (cm${}^{-1}$)	T2 Peak Area (%)	Assignment [3,5,14]
1578	10.2 ± 0.1	1582	4.0 ± 0.1 **	-	-	
1590	10.2 ± 0.1	1594	19.5 ± 0.2 **	1587	9.3 ± 0.2 **	
1602	10.3 ± 0.1	1605	14.4 ± 0.1	1604	21.0 ± 0.4	
1618	10.6 ± 0.1	1616	6.8 ± 0.1	1617	11.6 ± 0.1	Amide I (3${}_{10}$-helix; β-sheet)
1627	9.0 ± 0.1	1627	2.5 ± 0.1 **	1627	7.8 ± 0.2 *	
-	-	1632	6.5 ± 0.1	-	-	
1637	6.0 ± 0.1	1636	7.0 ± 0.2	1637	4.6 ± 0.1	
1644	8.8 ± 0.1	1642	7.0 ± 0.1	1645	8.5 ± 0.1	Amide I (α-helix)
1654	8.8 ± 0.1	1654	12.4 ± 0.1 *	1653	7.6 ± 0.1	Random coil
1665	24.0 ± 0.1	1661	6.2 ± 0.1 **	1667	29.3 ± 0.2 **	Amide I (β-turn)
1675	2.4 ± 0.1	1672	13.7 ± 0.1 **	1676	0.3 ± 0.1 **	Amide I (β-sheet)

The significance level was denoted as * *p* < 0.05, ** *p* < 0.01, and obtained by comparing T1/T0 and T2/T1 groups, respectively.

**Table 2:** Wavenumber position and relative peak areas of the main modes of the Amide I μ-FTIR spectral region of GCF and their assignment. The GCF were collected before starting the orthodontic process (T0 data) and after 2 days of treatment (T1 data), 7 days of treatment (T2 data), and 14 days of treatment (T3). The peak area percentage was evaluated by a fitting procedure, assuming the Amide I band area equal to 1. The values are shown as ‘mean ± standard deviation’ for each group. Bold values refer to Gaussian component, while normal font values refer to Lorentzian components. For comparison, the data reported in Ref. [16] are indicated.

T0	T0	T1	T1	T2	T2	T3	T3	Ref. [16]	Assignment
Modes (cm${}^{-1}$)	Peak Area (%)	Modes (cm${}^{-1}$)	Peak Area (%)	Modes (cm${}^{-1}$)	Peak Area (%)	Modes (cm${}^{-1}$)	Peak Area (%)	Peak Area (%)	[5,28]
**1634**	6.2 ± 0.1	-	-	**1634**	23.5 ± 0.1	1640	48.7 ± 0.1 **	37 ${}^{‡}$	β-sheet
**1644**	31.7 ± 0.2	1644	60.8 ± 0.7 *	**1644**	12.6 ± 0.1 **	**1646**	2.7 ± 0.1 **	20	disorder
**1655**	9.2 ± 0.1	**1656**	19.1 ± 0.4 *	**1655**	22.0 ± 0.1 *	**1659**	19.4 ± 0.4	27	α-helix
1665	26.4 ± 0.1	-	-	-	-	**1668**	3.1 ± 0.1	16	β-turn
1675	26.5 ± 0.1	**1673**	20.1 ± 0.1 **	**1671**	41.9 ± 0.1 **	1678	26.1 ± 0.2 **	-	β-sheet

The significance level was denoted as * *p* < 0.05, ** *p* < 0.01, and obtained by comparing T1/T0. T2/T1, and T3/T2 groups, respectively. ‡ The value refers to the total area of *β*-sheet components.

## Data Availability

The data are available on request.

## Abbreviations

The following abbreviations are used in this manuscript:

DWT	Direct Wavelet Transform
FTIR	Fourier Transform InfraRed spectroscopy
GCF	Gingival Crevicular Fluid
GNP	gold nano particles
μ-FTIR	Fourier Transform InfraRed micro-spectroscopy
μ-RS	micro-Raman spectroscopy
OTM	orthodontic tooth movement
SERS	surface enhanced Raman spectroscopy
SNV	standard normal variate
